# FPGA-Based Fused Smart Sensor for Dynamic and Vibration Parameter Extraction in Industrial Robot Links

**DOI:** 10.3390/s100404114

**Published:** 2010-04-26

**Authors:** Carlos Rodriguez-Donate, Luis Morales-Velazquez, Roque Alfredo Osornio-Rios, Gilberto Herrera-Ruiz, Rene de Jesus Romero-Troncoso

**Affiliations:** 1 HSPdigital – CA Mecatronica, Facultad de Ingenieria, Universidad Autonoma de Queretaro, Campus San Juan del Rio, Rio Moctezuma 249, 76807 San Juan del Rio, Qro., Mexico; E-Mails: cdonate@hspdigital.org (C.R.-D.); lmorales@hspdigital.org (L.M.-V.); raosornio@hspdigital.org (R.A.O.-R.); 2 Facultad de Ingenieria, Universidad Autonoma de Queretaro, Cerro de las Campanas s/n, 76010 Queretaro, Qro., Mexico; E-Mail: gherrera@uaq.mx; 3 HSPdigital – CA Telematica, DICIS, Universidad de Guanajuato, Carr. Salamanca-Valle km 3.5+1.8, Palo Blanco, 36700 Salamanca, Gto., Mexico

**Keywords:** smart sensor, motion dynamics, vibrations, accelerometer, FPGA

## Abstract

Intelligent robotics demands the integration of smart sensors that allow the controller to efficiently measure physical quantities. Industrial manipulator robots require a constant monitoring of several parameters such as motion dynamics, inclination, and vibration. This work presents a novel smart sensor to estimate motion dynamics, inclination, and vibration parameters on industrial manipulator robot links based on two primary sensors: an encoder and a triaxial accelerometer. The proposed smart sensor implements a new methodology based on an oversampling technique, averaging decimation filters, FIR filters, finite differences and linear interpolation to estimate the interest parameters, which are computed online utilizing digital hardware signal processing based on field programmable gate arrays (FPGA).

## 1. Introduction

Intelligent robotics, as defined by Lopez-Juarez, *et al.* [1], demands the integration of smart sensors [2,3] that allow the controller to efficiently measure physical quantities. Communication and data processing functionalities are two of the most important features in smart sensors [3], but data fusion is also desirable. Industrial manipulator robots require constant monitoring of several variables and their fusion [4–6] such as: motion dynamics, inclination, and vibration; these variables inform about the machine wellness, highlighting the necessity of a specialized smart sensor that provides sufficient information to evaluate the robot performance. This work is focused on the extraction of several parameters from the mentioned physical variables, related to a single axis industrial robot arm. Motion dynamics is defined as the time-dependent profiles for position, velocity, acceleration, and jerk [7] in a servomotor, and determines the motion trajectory of a single axis robotic arm to reach a specific position and orientation based on a motion controller that uses these profiles as reference. On the other hand, the robot inclination is related to the spatial orientation of the physical sensor, where angular position, velocity, and acceleration on each robot link can be inferred. In addition, during the arm motion, vibrations are generated mainly due to friction, gearing, joint wear, *etc*.; these vibrations are undesired movements that reflect potential failures or improper operating conditions, making necessary their continuous monitoring to detect possible problems. Summarizing, it is desirable to have a single system able to provide all the aforementioned parameters from each robot link.

Current literature points out that the encoder in servomotors [4,8–10] and the accelerometer [11–14] are two of the most widely used sensors to monitor motion dynamics and vibrations on computerized numeric control (CNC) machines and robotic manipulator arms. Conversely, in industry, automation demands the integration of smart sensors [2,3] and control drivers in an open-architecture fashion [1,15]. Motion dynamics has been estimated from an incremental optical encoder in [9,10] where position, velocity, acceleration, and jerk parameters are successfully obtained, but they do not present the information of vibrations nor inclination. The use of accelerometers is well established to obtain kinematics parameters [8,16–18] or to measure vibrations [19], but there are no reported works that cover a broad parameter spectrum. The acceleration signal in an accelerometer contains merged information from the inclination with respect to gravity and about vibrations; therefore, a separation of these parameters is desirable for further kinematics and vibration monitoring. The extraction of vibrations allows failure detection [20] and some intelligent sensors have been proposed to perform this task [14]. Accelerometers have been also included in servo control loops [12]. By analyzing vibrations in combination with force sensors, contact forces are measured and calibrated [13], manifesting the relevance of a proper separation of vibration signals from the raw accelerometer measurement. Moreover, static acceleration indicates the inclination of the accelerometer with respect to gravity and by taking these signals, kinematics calibration in a manipulator arm can also be performed [11]. Inclination parameters have been investigated utilizing an accelerometer as primary sensor [21]. Furthermore, an integrated approach utilizing an encoder and an accelerometer has been presented to accurately estimate velocity [8]. Additionally, sensors are becoming more intelligent by integrating signal conditioning, processing units, communication protocols, among other features [3]. Therefore, the development of a smart sensor that integrates data fusion of motion dynamics, inclination, and vibration parameters is considered an essential move towards intelligent-robotics.

This work presents a novel smart sensor that extracts motion dynamics and inclination parameters along with the separation of vibration information from a single link in industrial robots, based on the fusion of two primary sensors: an optical incremental encoder and a triaxial accelerometer. Motion dynamics is estimated from the encoder measurement to give position, velocity, acceleration, and jerk; whereas vibrations and inclination are separated from the accelerometer signal, for providing angular position, velocity, acceleration, and vibrations. Estimated parameters are computed online utilizing digital hardware signal processing techniques such as digital filtering, interpolation, finite differences, among others. These computer-intensive processing algorithms are implemented in a field programmable gate array (FPGA) for a smart sensor approach by integrating hardware signal processing and data communication in an embedded system.

## 2. Background

This section establishes the relationship among the estimated parameters on a robotics application, where the encoder gives information regarding motion dynamics and the accelerometer gives both inclination and vibration information.

### 2.1. Motion Dynamics

**Figure 1.**
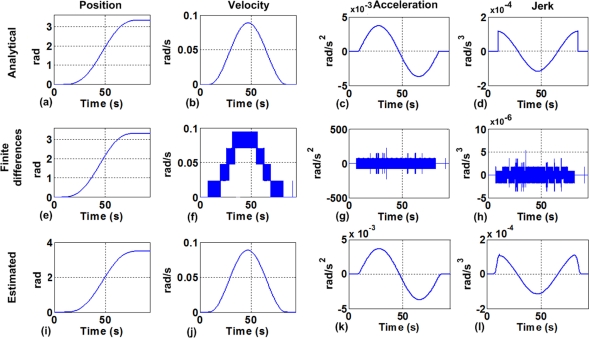
Analytical polynomial-motion profile: **(a)** position, **(b)** velocity, **(c)** acceleration, **(d)** jerk; recursive finite differentiation of the encoder: **(e)** position, **(f)** velocity, **(g)** acceleration, and **(h)** jerk; estimated motion profile with decimation: **(i)** position, **(j)** velocity, **(k)** acceleration, **(l)** jerk.

Motion in a manipulator arm is conducted by the motion controller that applies a profile to perform smooth movements to the end effector. By taking the position feedback signal (*p*) from the servo control loop, velocity (*v*), acceleration (*a*), and jerk (*j*) can be estimated. Figure 1 shows an example of a polynomial profile comparing the analytical motion dynamics (Figure 1a, 1b, 1c, and 1d) against the motion dynamics obtained using recursive finite differentiation (Figure 1e, 1f, 1g, and 1h). In the case of using finite differentiation, quantization noise overwhelms the signal, making some filtering necessary. This differentiation and filtering stage can be performed by a combination of an averaging decimation filter [22], a finite difference stage, and a linear interpolation stage to estimate motion dynamic parameters (*p*, *v*, *a*, and *j*) as shown in Figure 1i, 1j, 1k, and 1l.

### 2.2. Inclination

**Figure 2.**
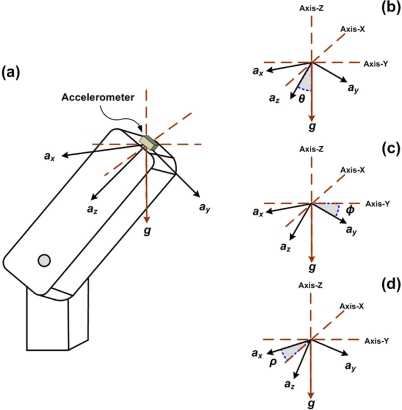
Triaxial accelerometer location on the robot arm, showing inclination angles with respect to gravity.

From the accelerations provided by the accelerometer (*a*_x_, *a*_y_, and *a*_z_), inclination angles pitch (*ρ*), roll (*φ*), and yaw (*θ)* are calculated. Figure 2 shows the diagram of a single axis robot arm depicting the triaxial accelerometer location, where *ρ* represents the inclination with respect to the X-axis, *φ* represents the inclination with respect to the Y-axis, and *θ* is the inclination with respect to the Z-axis.

The inclination is calculated from the acceleration provided by the accelerometer as stated by (1–3) taken from [23]. These equations assume that original signals (*a*_x_, *a*_y_, and *a*_z_) are noise-free, which is unrealistic, requiring filtering.

(1) $$\rho ={\text{tan}}^{-1}\left(\frac{a_{x}}{\sqrt{a_{y}^{2}+a_{z}^{2}}}\right)$$

(2) $$\phi ={\text{tan}}^{-1}\left(\frac{a_{y}}{\sqrt{a_{x}^{2}+a_{z}^{2}}}\right)$$

(3) $$\theta ={\text{tan}}^{-1}\left(\frac{\sqrt{a_{x}^{2}+a_{y}^{2}}}{a_{z}}\right)$$

### 2.3. Vibrations

The accelerometer provides merged information about vibration and inclination that must be separated; given that the inclination signal is principally low frequency [24] whereas vibration is high frequency [25], they can be separated with properly tuned filters. To reduce the noise and effectively extract the vibration signal, a decimation process followed by a high-pass filter is used to separate the three signals; this technique reduces the noise from the original signal.

## 3. Smart Sensor Methodology

**Figure 3.**
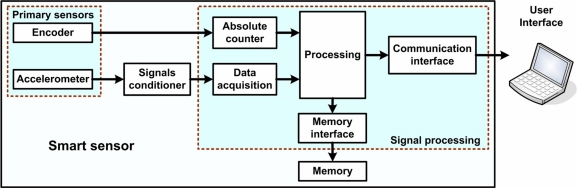
An overview of the architecture of the proposed smart sensor.

The proposed smart sensor is implemented in three stages: primary sensors, signal conditioner, and signal processing; Figure 3 shows the overall architecture for the proposed smart sensor. At the primary sensors stage the physical quantities are sensed, at the signal conditioning stage the accelerometer signal is amplified, and at the signal processing stage parameters are estimated. For each sampling period, the encoder measures the relative servomotor shaft position and gives two quadrature signals; the first step is to decode the absolute position of the servomotor shaft on the effector motions, then parameter estimation can be performed. The accelerometer reads the acceleration analog signals and then the signal conditioner amplifies these signals to pass them to the analog-to-digital converter, digitally normalized (m/s^2^). Once the sample is taken, the signal processing is started; at the end of this process the estimated parameters are stored in the memory via the memory interface; finally the user is able to request the desired information from the smart sensor via the communication interface.

**Figure 4.**
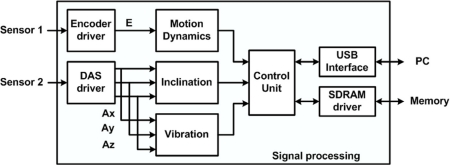
Smart sensor methodology showing the main blocks in the system.

Most of the proposed smart sensor functionality is performed by the digital signal processor inside the smart sensor, depicted in Figure 4. The motion dynamics block processes the encoder position signal (*E*) to obtain the motion dynamics parameters *p*, *v*, *a*, and *j*. The inclination block takes the acceleration from each axis (*A_x_*, *A_y_*, and *A_z_*) to calculate the inclination angles *ρ, φ ,* and *θ*. The vibration block separates the vibration signals from the inclination for further analysis. The control unit synchronizes the resulting parameters to maintain the correspondence among the signals; these parameters are then stored into an external synchronous dynamic random access memory (SDRAM) or sent via USB interface to the user PC.

The signal processing unit of the smart sensor needs to make intensive computations for obtaining motion dynamics, inclination, and vibration data from three acceleration signals and the encoder input. In order to properly achieve the required processing performance, an FPGA device is the most suited option for implementation thanks to the reconfigurability and parallelism features of these devices [25].

### 3.1. Motion Dynamics Methodology

**Figure 5.**
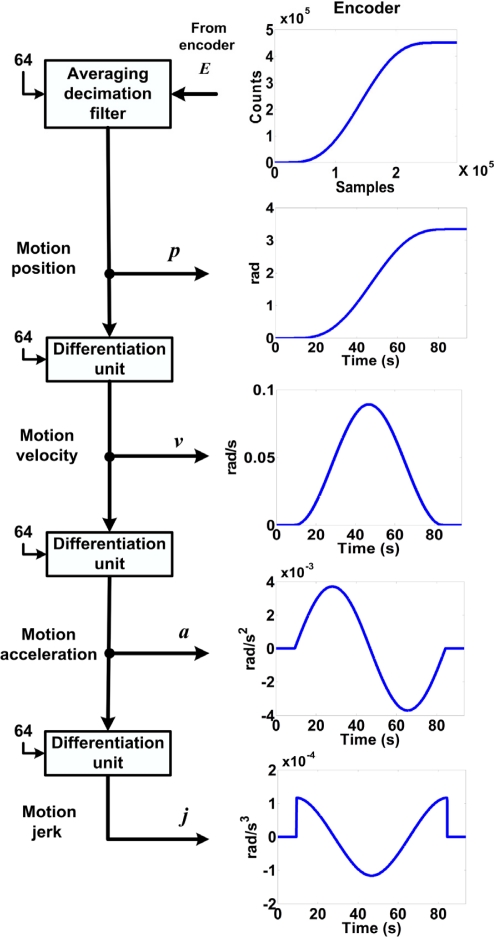
The motion dynamics methodology.

The motion dynamics methodology is shown in Figure 5. Since the primary sensor in this module is the encoder, 64-times oversampling is applied, requiring a decimation factor of 64 that is also applied to a differentiation unit described below. This figure also shows the process used to estimate the motion profile using only the encoder signal by cascading three low noise differentiation units to obtain *p*, *v*, *a*, and *j*.

The averaging decimation filter shown in Figure 5 is used for filtering the encoder signal in order to reduce the quantization noise as stated in (4) and exposed by Rangel-Magdaleno *et al*. [22] using an oversampling technique, where *E* is the encoder input signal, *p* the decimated position, *k* the discrete-time index, and *i* the decimation index.

(4) $$p\left(\frac{k}{64}\right)=\frac{1}{64}\sum_{i=0}^{63}E(k-i)$$

The differentiation unit performs a low-noise difference over *p* utilizing a finite difference operation defined in (5) to obtain *p_F_*. An averaging decimation filter is then applied to the differentiated signal as stated in (6) to obtain *p_D_*, and a linear interpolation using (7) to preserve the sampling rate and obtain the estimated velocity *v*. To estimate *a* and *j* an equivalent process is followed, recursively.

(5) $$p_{F}(k)=p(k)-p(k-1)$$

(6) $$p_{D}\left(\frac{k}{64}\right)=\frac{1}{64}\sum_{i=0}^{63}P_{F}(k-i)$$

(7) $$\begin{array}{ll} v(64\cdot k+i)=p_{D}(k-1)+\frac{1}{64}[p_{D}(k)-p_{D}(k-1)]\cdot i; & i=0,1,\ldots 63 \end{array}$$

### 3.2. Inclination Methodology

**Figure 6.**
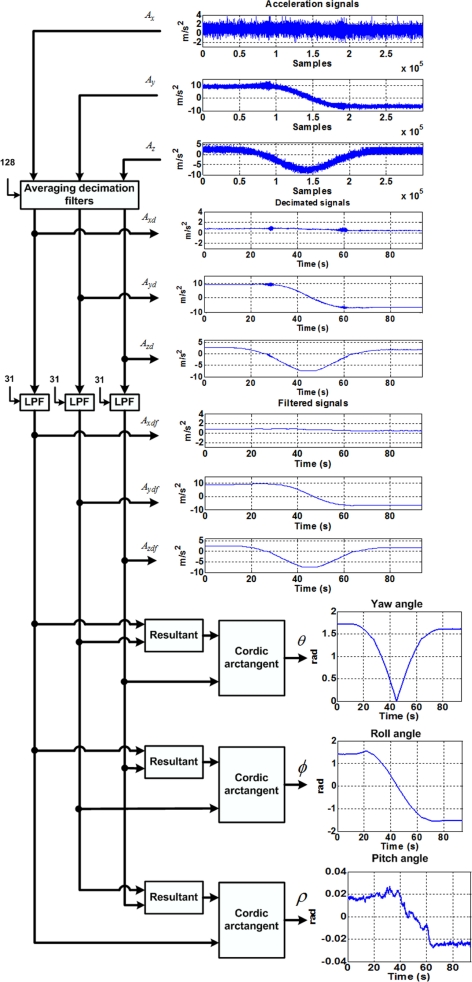
Methodology to estimate inclination angles from accelerometer signals.

The process for estimating inclination parameters is depicted in Figure 6. The raw triaxial acceleration signals are filtered by averaging decimation filters of the 128th order, as stated in (8), where subscript *s* represents the accelerometer axis (*x*, *y*, or *z*). Then, to separate inclination signals from vibration, a 32nd order low-pass FIR filter is applied to the decimated signals resulting in the inclination signals (*A_xdf_*, *A_ydf_*, and *A_zdf_*). To calculate inclination angles from the acceleration signals, it is necessary to compute the inverse tangent function; this is performed using the CORDIC algorithm [27].

(8) $$A_{\mathrm{sd}}\left(\frac{k}{128}\right)=\frac{1}{128}\sum_{i=0}^{127}A_{s}(k-i)$$

### 3.3. Vibration Methodology

**Figure 7.**
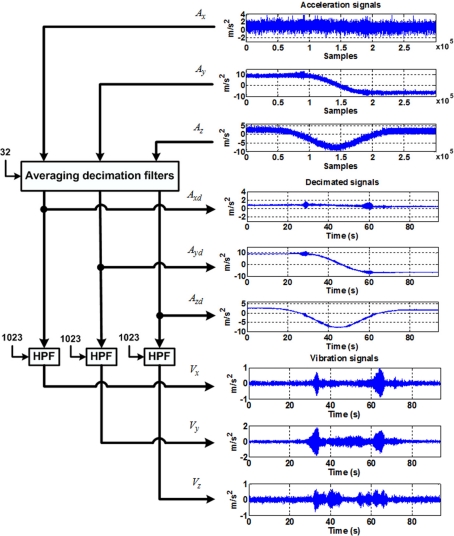
Vibration-parameters estimation methodology used to separate vibrations from the original accelerometer measurement.

To extract the vibration components from the raw accelerometer signals they are first filtered using a 32nd order averaging decimated filter as depicted in Figure 7; after this, the decimated signals (*A_xd_*, *A_yd_*, and *A_zd_*) are passed through a 1024th order high-pass FIR filter to isolate the vibration signals (*V_x_*, *V_y_*, and *V_z_*).

## 4. Experimental Results

In this section, the experimental validation of the proposed smart sensor is presented. The online parameter estimation was performed during a single arm movement in the second effector.

### 4.1. Experimental Setup

**Figure 8.**
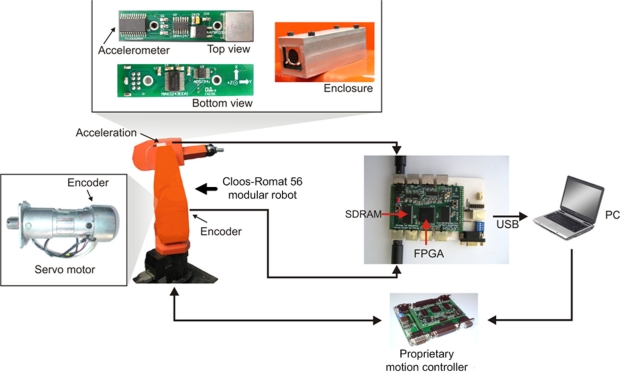
Experimental setup used for experimentation, showing the Cloos-Romat 56 modular robot, the two primary sensors, the FPGA processing unit, the proprietary motion controller, and the user PC.

The experimental setup consists on the instrumentation of a single axis of a 6-degree of freedom Cloos-Romat 56 modular robot, a triaxial accelerometer LIS3L02AS4 [28] with a signal bandwidth of 750 Hz, and a sampling frequency of 3.2 kHz at the three-channel on-board data acquisition system; a proprietary Spartan 3E XC3S1600E FPGA platform running at 48 MHz as the smart sensor processing unit, a proprietary motion controller, and a user PC, as shown in Figure 8. Processing units were implemented in the VHSIC hardware description language (VHDL) under the Xilinx ISE Design Suite version 11, since it has being extensively used to develop smart sensors [29,30]. Table 1 summarizes the resource usage of the FPGA after compilation.

The experiment consists of a single-axis movement of the second effector arm using the proprietary motion controller by applying a 7th order polynomial motion profile [7] during 90 seconds and extracting in real-time the required parameters that are stored in SDRAM and then sent to the user PC.

### 4.2. Motion Dynamics Results

**Figure 9.**
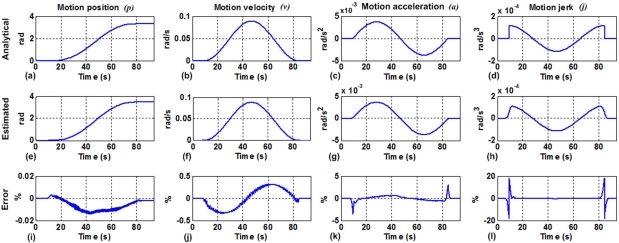
Motion dynamics results: **(a)** analytical position profile, **(e)** estimated position profile, **(i)** position error, **(b)** analytical velocity profile, **(f)** estimated velocity profile, **(j)** velocity error, **(c)** analytical acceleration profile, **(g)** estimated acceleration profile, (k) acceleration error, **(d)** analytical jerk profile, **(h)** estimated jerk profile, and **(l)** jerk error.

Figure 9 shows the analytical profiles that were obtained from the motion controller, whereas the estimated ones are computed by the smart sensor for the motion dynamics estimation. The upper row is the profile applied to the motion controller to conduct the arm movement; the middle row is the estimated profile obtained from the encoder measurement by applying the proposed methodology, and the bottom row is the computed error between these signals. For all parameters the shape of the analytical profile is well fitted, and the error between the analytical and the estimated signals is quantitatively calculated. The estimated position profile in Figure 9e is very similar to the analytical one in Figure 9a, having an absolute error below 0.01%. The estimated velocity in Figure 9f fits the analytical profile of Figure 9b with an overall absolute error below 0.5%. The estimated acceleration of Figure 9g is very similar in shape to the analytical one in Figure 9c with an absolute error below 5%. The estimated jerk on Figure 9h also shows a good fitting to the analytical one in Figure 9d, but the error is increased mainly because the initial and final discontinuities in the jerk shape, having an absolute error below 20% in those sections and below 5% in the middle section.

### 4.3. Inclination Results

**Figure 10.**
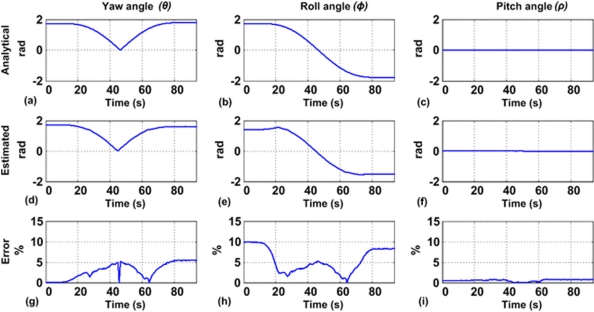
Inclination estimated results: (a) analytical yaw, (b) analytical roll, (c) analytical pitch, (d) estimated yaw, (e) estimated roll, (f) estimated pitch, (g) yaw relative error (h) roll relative error, and (i) pitch relative error.

The inclination results from the accelerometer measurements follow the methodology described in section 3.2. The estimated inclination angles shown in Figure 10 were computed using the same trajectory used in the previous section for motion dynamics. Analytical inclination angles were calculated from the motion position profile. The analytical yaw in Figure 10a, compared with the estimated yaw in Figure 10d, gives a maximum error of 6% in Figure 10g. Analytical roll of Figure 10b is similar to the estimated roll of Figure 10e with an error below 10% on Figure 10h. Finally, analytical pitch angle of Figure 10c, compared with the estimated pitch of Figure 10f, gives an error below 1% on Figure 10i. As presented in Figure 10, analytical and estimated angles are very similar in shape with an overall error below 10%.

### 4.4. Vibration Results

**Figure 11.**
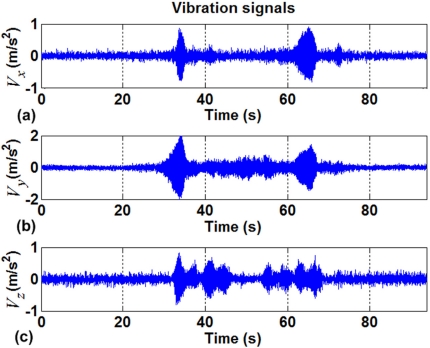
Vibration information separated from inclination at the accelerometer **(a)** X-axis, **(b)** Y-axis, and **(c)** Z-axis.

The vibration separation performed by the smart sensor is shown in Figure 11; this figure shows the three axis vibration signals, separated from the accelerometer measurements. The obtained vibration signals are identified with respect to time and the motion profile shape because they were taken using the same primary sensor in the system. Hence it is possible to relate the inclination results with vibration results and, in a further analysis, to identify gearing or friction problems.

### 4.5. Discussion

Experimental results using the proposed smart sensor present the obtained motion dynamics, inclination angles and vibrations over a single axis robot link. In the case of motion dynamics, position, velocity, and acceleration were obtained having an overall error below 5%, and below 20% for jerk estimation. Inclination angles were successfully separated from vibrations and the error between the analytical and the estimated values were below 10% for all angles. The separated vibration signals from the smart sensor do not contain information on the inclination and the user can further process the information for monitoring and diagnosis purposes.

The parameter extraction is possible in the developed smart sensor thanks to the FPGA parallelism and reconfigurability. These features allow the hardware-processing unit of the smart sensor to efficiently perform the related smart operations such as hardware signal processing (data acquisition drivers, CORDIC arc tangent estimation, and oversampling, decimation, FIR, and interpolation filtering), data storage, and data communication. The developed sensor can be utilized in many different areas with a variety of applications; Table 2 presents several application examples on high-impact researches where the developed smart sensor can be utilized.

## 5. Conclusions

This work proposes a new smart sensor to simultaneously obtain several parameters related to motion dynamics and inclination, along with the separation of the vibration information using two primary sensors: an encoder and a triaxial accelerometer on a single link of industrial robots. Results on motion dynamics and inclination estimations show the effectiveness of this smart sensor that integrates data fusion among primary sensor data. The vibration signal result, provided by the smart sensor, does not contain the inclination information and it contains the vibration information only, which can be further processed for monitoring and diagnosis purposes. Furthermore, the proposed smart sensor was implemented in a low-cost FPGA where hardware signal processing units compute in parallel the parameters and integrate the necessary modules with a system on-a-chip approach. The developed sensor provides the information of a single robot link, but one smart sensor can be placed on each robot link and combine the obtained information from all of them to estimate other parameters such as direct kinematics or make assessments on the robot wellness; however, this is beyond the scope of the present research and it is left for future work.

## Figures and Tables

**Table 1.** FPGA resource usage.

1.6 million-gate Xilinx Spartan 3E FPGA: XC3S1600E			
Element	Used	Available	Percentage %
Slices	2027	4752	13
Slice Flip-flops	2158	2950	47
4-input LUTs	2719	2959	49
Block RAMs	6	36	17
Multipliers	25	36	70

**Table 2.** Smart sensor parameter application areas.

Primary sensor	Parameter group	Parameter	Applications
Encoder	Motion dynamics	Position	Positioning [10,31]
		Velocity	Control loop [32,33]
		Acceleration	Control loop [8]
		Jerk	Machine wear [7,31]
Accelerometer	Inclination	Angular position	Calibration [11], positioning [16,18]
		Angular velocity	Control loop [17,18]
		Angular acceleration	Torque control [12,13]
	Vibrations	Vibration	Failure detection [14]
